# Combining a variable‐centered and a person-centered analytical approach to caregiving burden – a holistic approach

**DOI:** 10.1186/s12877-021-02238-2

**Published:** 2021-04-30

**Authors:** Qi Yuan, Gregory Tee Hng Tan, Peizhi Wang, Fiona Devi, Richard Goveas, Harish Magadi, Li Ling Ng, Siow Ann Chong, Mythily Subramaniam

**Affiliations:** 1 Research Division, Institute of Mental Health, Buangkok Green Medical Park, 10 Buangkok View, 539747 Singapore, Singapore; 2 Department of Geriatric Psychiatry, Institute of Mental Health, Singapore, Singapore; 3 Department of Psychological Medicine, Changi General Hospital, Singapore, Singapore

**Keywords:** Informal caregivers, Dementia, Caregiving burden, Factor analysis, Latent class analysis

## Abstract

**Background:**

Informal caregivers of persons with dementia often experience elevated levels of caregiving burden. However, existing studies tend to use a variable-centered approach to explore it. This study aims to understand the caregiving burden of informal caregivers of persons with dementia in Singapore through a combination of variable-centered and person-centered analytical approaches, and explore the correlates of identified factors and latent classes of caregiving burden.

**Methods:**

Zarit Burden Interview was used to gauge the caregiving burden of 282 primary informal caregivers of persons with dementia recruited through convenience sampling in Singapore. Factor analysis and latent class analysis were conducted to identify the latent factors and the latent classes of Zarit Burden Interview, followed by multiple linear regression and multinomial logistic regression to explore their significant correlates.

**Results:**

The analyses suggested a 17-item 3-factor structure for Zarit burden interview and three mutually exclusive caregiving burden classes. Regression analyses found that caregiving related variables especially care recipients’ memory and behaviour problems were correlated with both the factors and latent classes of caregiving burden.

**Conclusions:**

The combination of these two approaches suggests that caregivers experiencing higher burden on one domain are likely to experience higher burden on the other two domains. This further supports the point that more attention should be given to caregivers who experience an overall high burden. Future research could explore the generalizability of our findings among caregivers elsewhere and explore the type of support needed by caregivers, especially those experiencing high burden.

## Background

The World Health Organization defines dementia as a syndrome in which there is deterioration in memory, thinking, behavior and the ability to perform everyday activities [1]. Such deterioration often leads to the high dependence among persons with dementia (PWD) and their subsequent need for care, especially from informal caregivers such as family members [2]. Taking care of PWD is usually very challenging for their caregivers [3]. Potential reasons could be the considerable investment of their time in caregiving [4, 5], and the conflicts between their social and family needs and caregiving [6, 7]. Caregiving burden refers to the extent to which caregiver perceives that caregiving has had an adverse effect on their emotional, social, financial, physical and spiritual function [8], and it might lead to negative consequences such as depression if caregivers are not able to cope with these stressors. According to the literature, caregiving burden is usually positively associated with caregiver distress, anxiety and depression [9], indicating its importance on the mental well-being of informal dementia caregivers.

Caregiving burden is usually measured by self-report scales, with Zarit Burden Interview (ZBI) [10] being one of the most frequently used tools. This 22-item scale was first developed in 1980, and has been validated in various countries including Singapore [11]. Existing studies on ZBI are dominated by the use of variable-centered analytical approaches to extract the latent factors from the communality between measuring items. For instance, a study among informal caregiver of PWD in the United States identified a 3-factor structure of ZBI, including impact of caregiving on caregivers’ lives, guilt, and frustration/embarrassment [12]. Similarly, another study in UK also found a 3-factor structure of ZBI among informal dementia caregivers [13]. This approach was also used by researchers in Singapore to study caregiving burden, and they suggested a 4-factor structure of ZBI among local dementia caregiver [14, 15]. However, since one of the factors identified in the Singapore study only had two items [14], less than the recommended minimum three items per factor [16], it was thus suggested that further studies are necessary to identify a more stable factor structure of ZBI among dementia caregivers in Singapore.

One issue with the traditional variable-centered approach is that this approach focuses on explaining relationships between the variables of interest in a population [17]. It often assumes a homogeneity of the study sample thus overlooking the possibility that caregivers might experience different levels of burden under each domain of ZBI. On the other hand, researchers realized that this approach failed to capture the diverse nature of the sample and could lead to over-generalized conclusions regarding the population [18]. To overcome these limitations, a complementary person-centered analytical approach (i.e., latent class analysis [19]) is proposed in the current study, to explore the unobserved subgroups of caregiving burden (latent classes). This approach assumes an inherently heterogeneous sample [20, 21], and enables variables to be analyzed jointly (i.e., coping strategies among informal caregivers among the current study). As a result, a combination of these two approaches would provide complementary views and in the ideal case, binocular views [21] of caregiving burden among informal dementia caregivers. Similar analytical approaches have also been used among other studies such as on the structure of prejudice [22] and the relationship between childhood maltreatment and adult psychopathology [23]. By including a person-centered analytical approach, a more objective picture of caregiving burden would be obtained.

The current study aims (1) to understand caregiving burden through a combination of both variable-centered and person-centered analytical approaches among informal caregivers of PWD in Singapore; (2) to investigate the significant correlates of the identified latent factors and latent classes of ZBI; (3) to make comparisons of the correlates of ZBI across the two analytical approaches.

## Methods

### Participants and procedures

From Jan 2017 to Dec 2018, primary informal caregivers of PWD were recruited from the outpatient and satellite clinics of the Institute of Mental Health and a geriatric clinic in Changi General Hospital in Singapore. A recruitment flyer was also put up in a Voluntary Welfare Organization that serves local caregivers. Participants needed to meet the following eligibility criteria: (1) Singapore residents (including citizen and permanent residents); (2) aged 21 years old and above; (3) taking care of a patient who has been formally diagnosed with dementia; (4) and able to communicate in either English, Mandarin or Malay. Caregivers were excluded if they had difficulty understanding the informed consent or if they failed to visit the PWD on a weekly basis. Data from eligible participants were collected through interviewer-administered questionnaire to ensure their understandings over the items. The choice of the language for administration was based on participants’ own preference. In all, 282 caregivers were recruited. More information about the study can be found in earlier articles [24–26].

The study was approved by the National Healthcare Group Domain Specific Review Board in Singapore (reference number: 2016/00921).Written informed consent was obtained from all participants. All methods in the current study were carried out in accordance with relevant guidelines and regulations.

### Measurements

Caregiving burden was measured by the Zarit Burden Interview (ZBI) [10]. This scale consists of 22 items on the perception of caregiving, sample items include ‘do you feel that your relative asks for more help than he/she needs?’ and ‘do you feel angry when you are around your relatives?’ The response of each item was coded on a Likert scale from 0 (never) to 4 (nearly always). The total score was calculated by summing up the item scores, with higher scores indicating higher perceived caregiving burden. This scale has been used in Singapore before, with very good internal reliability (Cronbach’s alpha = 0.93) and good test-retest reliability (*r* = 0.89) [11]. In the current study, its internal reliability was 0.92.

Functional dependence of the PWD was measured by the Activities of Daily Living Scale (ADL) [27] and the Instrumental Activities of Daily Living Scale (IADL) [28]. The ADL has six items, measuring patient disability in six basic self-care activities (i.e., bathing, dressing, toileting, transfer, continence and feeding). The IADL includes eight items, and it covers eight other higher order self-care activities (i.e., ability to use the telephone, shopping, food preparation, housekeeping, laundry, mode of transportation, responsibility for own medication, and ability to handle finances). The internal reliability of ADL and IADL in the current study were 0.82 and 0.74 respectively.

The memory and behaviour problems of PWD (MBP) were assessed by the memory (7 items) and behavior disruption (8 items) domains of the Revised Memory and Behaviour Problems Checklist (RMBPC) [29]. It has been used in Singapore before and has shown good internal reliability for both the memory (Cronbach’s alpha = 0.87) and behaviour subscale (Cronbach’s alpha = 0.73) [30]. In the current study, the internal reliability was 0.65 for the memory subscale, and 0.71 for the behaviour subscale. These two subscales were summed up to formulate a single indicator on PWD’s memory and behaviour problems, and its internal reliability was 0.74.

Socio-demographic information including the caregiver’s age, gender, ethnicity, education level, marital status, employment status was collected. Caregiving related variables including relationship to the PWD, living arrangement with the PWD, having a domestic helper or not, support from known networks during the past month, weekly caregiving hours, caregiving duration, and self-rated health were also collected.

### Data analysis

We firstly conducted the descriptive analysis for socio-demographic and caregiving-related variables, with continuous data being presented as mean and standard deviation (SD) and categorical data as frequency and percentage. The variable-centered analytical approach was proposed as following: (1) search for factor structures of ZBI in the literature among informal dementia caregivers globally; (2) run confirmatory factor analyses (CFA) among the current sample with the identified factor structures of ZBI in the existent literature; (3) only if CFA (step 2) fails to confirm a suitable solution, exploratory factor analysis (EFA) would be run. CFA was performed with the ‘lavaan’ package under R software [31], and adjusted for categorical variables with the estimator of ‘Weighted Least Square Means and Variance Adjusted’ [32]. In the current study, an acceptable model was defined as 1) the comparative fit index (CFI) > 0.90; 2), the Tucker-Lewis index (TLI) > 0.90, and 3) the root mean square error of approximation (RMSEA) < 0.08 [33]. In the end, a revised ZBI was generated during this step, and it is more applicable for informal dementia caregivers in Singapore.

To ensure a seamless integration and comparison with the factor analysis results, the person-centered analytic approach (i.e. latent class analysis (LCA)) was conducted using PROC LCA in SAS 9.3 [34] with the items included in the revised ZBI finalized during the previous factor analysis. The Akaike Information Criteria (AIC) [35], the Bayesian Information Criteria (BIC) [36], the consistent Akaike Information Criteria (cAIC) [37] and the interpretability of competing solutions [38] were considered while selecting the model with the optimal number of latent classes. Low information criteria indicate better fitting. Similar to previous studies [26, 39, 40], interpretability was considered when information criteria contradicted; and a model with latent prevalence less than or equal to 10 % was considered as limited clinical relevance in the current study. Following the three-step of fitting an LCA [41], the latent class membership generated from the LCA were treated as observed variables in the follow-up regression analysis.

Lastly, multiple linear regression was then conducted to explore the significant correlates of each ZBI factor, and multinomial logistic regression was conducted to identify the correlates of ZBI latent classes. A two-sided p-value below 0.05 was considered as statistically significant for the multiple linear regression. For multinomial logistic regression, since two separate regressions were run by changing the reference level, its significance level was adjusted to 0.025 using Bonferroni correction. The descriptive and the regression analyses were all conducted using SAS 9.3.

## Results

The socio-demographic characteristics of the study sample are shown in Table 1. Participants had an average age of 55.7 years, with the majority being female (75.2 %), Chinese (83 %), and married/divorced/widowed (72 %). Only 31.6 % of them had an education level of degree or above, and more than half (57.1 %) were employed at the time of recruitment. More than half of the caregivers were daughters of PWD (55.3 %), followed by son-caregivers (17.0 %) and spousal-caregivers (15.3 %). Around three quarters (70.2 %) of the caregivers were living with the PWD, and more than half did not have a domestic helper (57.1 %) to help with the caregiving. Two-fifths of the participants reported that they have received formal support in caregiving during the past month, and rated their health as fair or poor. On average, the caregivers had taken care of the PWD for 52.4 months, providing average weekly caregiving of 55.0 h.

**Table 1:** Descriptive statistics of the study sample (*n* = 282)

	Frequency	Percentage (%)
**Gender**		
Male	70	24.8
Female	212	75.2
**Ethnicity**		
Chinese	234	83.0
Malay	29	10.3
Indian & others	19	6.7
**Education level**		
Secondary or below (include N/O level)	120	42.6
A level, polytechnic and other diploma	73	25.9
Degree or above	89	31.6
**Marital status**		
Single	79	28.0
Married/divorced/widowed	203	72.0
**Employment status**		
Unemployed/retired/housewife	121	42.9
Employed	161	57.1
**Relationship to the PWD**		
Spouse	43	15.3
Son	48	17.0
Daughter	156	55.3
Others	35	12.4
**Living arrangement**		
With PWD	198	70.2
Separated from PWD	84	29.8
**Domestic helper**		
Have	121	42.9
Don’t have	161	57.1
**Received formal support during past month**		
Yes	112	39.7
No	170	60.3
**Self-rated health**		
Fair or poor	113	40.1
Good or excellent	169	59.9
	**Mean**	**SD**
**Age (years)**	55.7	11.8
**Weekly caregiving hours**	55.0	53.0
**Caregiving duration (months)**	52.4	53.8
**ADL**	2.4	1.9
**IADL**	5.9	1.9
**MBP**	6.9	3.1

During the literature search, we found eight articles documenting different factor structures of ZBI among informal dementia caregivers [12–14, 42–46]. However, six out of the eight ZBI factor structures had at least one domain containing only two items [14, 42–46]. As such, they were all excluded from the test as they failed to meet the requirement of a minimum of three items per factor [16]. Hence, CFA was conducted only for the one-dimension structure with all 22 items of ZBI (model 1), and the 3-factor structure from a UK sample [13] (model 2), and another 3-factor structure from a US sample [12] (model 3). The CFA results suggested that both model 1 and 3 had poor model fit, while model 2 had an acceptable model fit (RMSEA still shows mediocre fit [47]). In this case, the 3-factor structure from model 2 was selected, and the EFA was not conducted. The three factors were named the same as in the previous study, including factor 1 - impact on caregiver life (item 12, 2, 17, 11, 3, 22, 15, and 10), factor 2 - uncertainty over future (item 19, 16 and 7), and factor 3 - frustration/embarrassment (item 5, 9, 4, 13, 18 and 6). Please refer to Table 2 for the model fit indices.

**Table 2:** Model fit indices for CFA of ZBI

	Model 1	Model 2	Model 3
**Chi-square**	1427.66	343.36	821.28
**Degree of freedom**	209	116	132
**CFI**	0.819	0.962	0.886
**TIL**	0.800	0.955	0.868
**RMSEA**	0.144	0.084	0.136

Note: Model 1- one-dimension model with 22 ZBI items; Model 2–3-factor structure with 17 ZBI items [13]; Model 3–3-factor structure with 18 ZBI items [12]

**Fig. 1:**
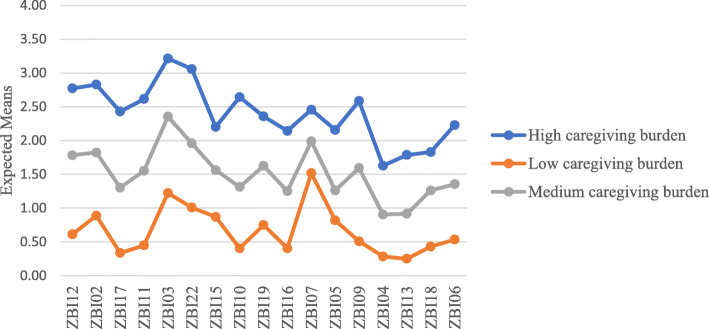
The caregiving burden pattern among informal dementia caregivers.

Latent class analysis was conducted based on the 17 items included in the revised ZBI from the above factor analysis. Table 3 shows the model fit indices of the latent class analysis. Both BIC and cAIC favors the 3-class solution while AIC favors the 5-class model. In this case, models from 3- to 5-class were all considered. Since one of the classes in the 5-class solution had a prevalence less than 10 % (i.e. 5.3 %), this solution was excluded. After considering the interpretability, the 3-class model was selected in the end, suggesting that the local dementia caregivers have three different latent classes of caregiving burden. Figure 1 plotted the different patterns of caregiving burden among informal dementia caregivers. As seen from this figure, these three classes were named as ‘high caregiving burden’ group – class 1 (*n* = 70, 25 %); ‘medium caregiving burden’ group – class 3 (*n* = 96, 34 %); and ‘low caregiving burden’ group – class 2 (*n* = 116, 41 %).

**Table 3:** Comparisons of model fit indices for fitted LCA models

Number of classes	Log-likelihood	AIC	BIC	cAIC	Entropy
3	-5798.11	8831.73	**9581.96**	**9787.96**	0.94
4	-5679.18	8731.87	9733.39	10008.39	0.94
5	-5598.23	**8707.98**	9960.8	10304.8	0.95

The multiple linear regression suggested that impact on caregiver life was positively associated with PWD’s ADL (β = 0.61, 95 %CI 0.05 to 1.18, *p* = 0.034) and MBP (β = 0.79, 95 %CI 0.53 to 1.05, *p* < 0.001), and caregiver’s weekly caregiving hours (β = 0.03, 95 %CI 0.01 to 0.05, *p* < 0.001). It was negatively associated with being of Malay ethnicity (vs. Chinese ethnicity, β=-3.56, 95 %CI -6.25 to -0.88, *p* = 0.010) and self-rated health status of good or excellent (vs. fair or poor, β=-2.95, 95 %CI -4.58 to -1.32, *p* < 0.001). Uncertainty over future was positively associated with PWD’s MBP (β = 0.23, 95 %CI 0.13 to 0.34, *p* < 0.001), and negatively associated with age (β=-0.06, 95 %CI -0.09 to -0.02, *p* = 0.003), non-spouse relationships to the PWD (son vs. spouse - β=-1.68, 95 %CI -3.25 to -0.10, *p* = 0.037; daughter vs. spouse - β=-1.88, 95 %CI -3.22 to -0.54, *p* = 0.006; others vs. spouse - β=-2.35, 95 %CI -3.84 to -0.85, *p* = 0.002), and self-rated health of good or excellent (β=-0.99, 95 %CI -1.66 to -0.32, *p* = 0.004). Frustration/embarrassment was positively associated with MBP (β = 0.52, 95 %CI 0.33 to 0.71, *p* < 0.001), and negatively associated with age (β=-0.07, 95 %CI -0.13 to -0.003, *p* = 0.041). Please refer to Table 4 for the details.

**Table 4:** Multiple linear regression of factors of ZBI

	Impact on Caregiver life				Uncertainty over future				Frustration/embarrassment			
	**β**	**95 % CI**		**p**	**β**	**95 % CI**		**p**	**β**	**95 % CI**		**p**
**Age**	-0.06	-0.15	0.03	0.200	-0.06	-0.09	-0.02	**0.003**	-0.07	-0.13	-0.003	**0.041**
**Gender**												
Male	-1.25	-4.63	2.12	0.466	-0.58	-1.97	0.80	0.408	-0.43	-2.83	1.97	0.725
Female	Ref				Ref				Ref			
**Ethnicity**												
Chinese	Ref				Ref				Ref			
Malay	-3.56	-6.25	-0.88	**0.010**	-0.55	-1.65	0.55	0.325	-1.64	-3.56	0.27	0.091
Indian & others	0.27	-2.87	3.40	0.868	0.33	-0.95	1.62	0.610	1.33	-0.90	3.56	0.242
**Education level**												
Secondary or below (include N/O level)	-1.34	-3.27	0.60	0.175	-0.41	-1.20	0.39	0.313	-0.42	-1.80	0.96	0.550
A level, polytechnic and other diploma	-1.43	-3.45	0.59	0.165	-0.49	-1.32	0.34	0.245	-0.82	-2.26	0.62	0.265
Degree or above	Ref				Ref				Ref			
**Marital status**												
Single	Ref				Ref				Ref			
Married/divorced/widowed	-1.38	-3.24	0.47	0.143	-0.37	-1.13	0.39	0.339	-0.52	-1.84	0.80	0.439
**Employment status**												
Unemployed/retired/housewife	Ref				Ref				Ref			
Employed	0.06	-1.74	1.86	0.947	-0.63	-1.37	0.10	0.092	-0.85	-2.12	0.43	0.194
**Relationship to the PWD**												
Spouse	Ref				Ref				Ref			
Son	-0.06	-3.90	3.78	0.976	-1.68	-3.25	-0.10	**0.037**	-0.89	-3.62	1.85	0.524
Daughter	-1.01	-4.28	2.26	0.544	-1.88	-3.22	-0.54	**0.006**	-0.29	-2.62	2.03	0.803
Others	-3.09	-6.73	0.56	0.097	-2.35	-3.84	-0.85	**0.002**	-1.46	-4.06	1.14	0.269
**Living arrangement**												
Living withith PWD	0.51	-1.50	2.51	0.618	-0.79	-1.61	0.04	0.061	0.91	-0.52	2.34	0.209
Living separately from PWD	Ref				Ref				Ref			
**Domestic helper**												
Have	-0.23	-2.00	1.55	0.803	0.47	-0.25	1.20	0.200	-0.22	-1.48	1.04	0.734
Don’t have	Ref				Ref				Ref			
**Received formal support during past month**												
Yes	Ref				Ref				Ref			
No	-1.58	-3.28	0.11	0.066	-0.14	-0.83	0.55	0.691	-0.97	-2.18	0.23	0.114
**Self-rated health**												
Fair or poor	Ref				Ref				Ref			
Good or excellent	-2.95	-4.58	-1.32	**< 0.001**	-0.99	-1.66	-0.32	**0.004**	-0.97	-2.13	0.19	0.100
**Weekly caregiving hours**	0.03	0.01	0.05	**< 0.001**	-0.0001	-0.01	0.01	0.973	0.001	-0.01	0.01	0.826
**Caregiving duration (months)**	-0.01	-0.02	0.01	0.210	-0.0009	-0.01	0.01	0.769	0.001	-0.01	0.01	0.904
**ADL**	0.61	0.05	1.18	**0.034**	-0.03	-0.26	0.20	0.810	-0.01	-0.41	0.39	0.960
**IADL**	-0.12	-0.71	0.47	0.688	-0.02	-0.26	0.22	0.862	0.12	-0.30	0.53	0.588
**MBP**	0.79	0.53	1.05	**< 0.001**	0.23	0.13	0.34	**< 0.001**	0.52	0.33	0.71	**< 0.001**

The multinomial logistic regression found that caregivers caring for PWD with higher MBP was the only consistent factor correlating with higher odds of caregivers being in the higher burden groups (medium vs. low – odds ratio (OR) = 1.24, 95 % CI 1.11 to 1.39, *p* < 0.001; high vs. medium – OR = 1.17, 95 %CI 1.04 to 1.33, *p* = 0.012; high vs. low – OR = 1.46, 95 % CI 1.27 to 1.67, *p* < 0.001). Caregivers who reported having good or excellent health were less likely to be in the high burden group compared to the medium- (vs. fair or poor - OR = 0.42, 95 % CI 0.20 to 0.90, *p* = 0.025) and low burden groups (vs. fair or poor – OR = 0.35, 95 % CI 0.16 to 0.78, *p* = 0.010). Caregivers who reported higher weekly caregiving hours were more likely to be in the high caregiving burden group in comparison to the low caregiving burden group (OR = 1.01, 95 % CI 1.002 to 1.02, *p* = 0.022). Furthermore, less educated caregivers were less likely to be in the high burden group compared to the low burden group (secondary or below vs. degree or above - OR = 0.33, 95 % CI 0.13 to 0.86, *p* = 0.022). Caregivers caring for PWD with more ADLs were more likely to be in the high burden group compared to the low burden group (OR = 1.49, 95 % CI 1.12 to 1.99, *p* = 0.006). Details of the multinomial logistic regression are included in Table 5.

**Table 5:** Multinomial logistic regression of ZBI latent classes

	High caregiving burden group VS. low caregiving burden group				Medium caregiving burden group VS. low caregiving burden group				High caregiving burden group VS. medium caregiving burden group			
	**OR**	**95 % CI**		**p**	**OR**	**95 % CI**		**p**	**OR**	**95 % CI**		**p**
**Age**	0.97	0.93	1.02	0.234	0.99	0.96	1.03	0.611	0.98	0.94	1.02	0.413
**Gender**												
Male	0.55	0.10	2.95	0.487	0.17	0.03	0.93	0.041	3.26	0.42	25.16	0.257
Female	Ref				Ref				Ref			
**Ethnicity**												
Chinese	Ref				Ref				Ref			
Malay	0.40	0.11	1.53	0.181	0.53	0.19	1.50	0.231	0.76	0.21	2.79	0.679
Indian & others	2.79	0.69	11.18	0.149	0.64	0.17	2.52	0.527	4.32	0.96	19.43	0.056
**Education level**												
Secondary or below (include N/O level)	0.33	0.13	0.86	**0.022**	0.54	0.25	1.20	0.133	0.61	0.26	1.45	0.265
A level, polytechnic and other diploma	0.33	0.12	0.93	0.036	0.76	0.35	1.67	0.495	0.44	0.17	1.14	0.089
Degree or above	Ref				Ref				Ref			
**Marital status**												
Single	Ref				Ref				Ref			
Married/divorced/widowed	0.57	0.23	1.43	0.228	0.63	0.30	1.33	0.224	0.91	0.40	2.04	0.809
**Employment status**												
Unemployed/retired/housewife	Ref				Ref				Ref			
Employed	0.62	0.26	1.45	0.268	0.81	0.39	1.67	0.571	0.76	0.34	1.70	0.504
**Relationship to the PWD**												
Spouse	Ref				Ref				Ref			
Son	0.56	0.08	3.92	0.560	4.32	0.69	27.09	0.118	0.13	0.01	1.22	0.074
Daughter	0.61	0.12	3.02	0.543	0.85	0.23	3.23	0.813	0.71	0.16	3.29	0.666
Others	0.14	0.02	0.99	0.049	0.52	0.12	2.26	0.381	0.27	0.04	1.83	0.180
**Living arrangement**												
Living withith PWD	0.70	0.26	1.89	0.478	1.10	0.51	2.37	0.816	0.64	0.24	1.67	0.357
Living separately from PWD	Ref				Ref				Ref			
**Domestic helper**												
Have	1.60	0.66	3.88	0.302	1.13	0.56	2.29	0.733	1.41	0.61	3.26	0.420
Don’t have	Ref				Ref				Ref			
**Received formal support during past month**												
Yes	Ref				Ref				Ref			
No	0.52	0.23	1.16	0.110	0.87	0.44	1.70	0.678	0.59	0.28	1.28	0.183
**Self-rated health**												
Fair or poor	Ref				Ref				Ref			
Good or excellent	0.35	0.16	0.78	**0.010**	0.83	0.43	1.60	0.580	0.42	0.20	0.90	**0.025**
**Weekly caregiving hours**	1.01	1.002	1.02	**0.022**	1.001	0.99	1.01	0.772	1.01	1.001	1.02	0.029
**Caregiving duration (months)**	1.00	0.99	1.01	0.597	0.9980	0.99	1.00	0.403	1.00	0.99	1.01	0.895
**ADL**	1.49	1.12	1.99	**0.006**	1.19	0.95	1.49	0.123	1.25	0.96	1.63	0.102
**IADL**	0.85	0.63	1.15	0.285	1.02	0.81	1.29	0.871	0.83	0.62	1.12	0.221
**MBP**	1.46	1.27	1.67	**< 0.001**	1.24	1.11	1.39	**< 0.001**	1.17	1.04	1.33	**0.012**

## Discussion

The current study provides a holistic understanding of caregiving burden among informal dementia caregivers, including its factor structure and latent classes to capture the diverse nature of the sample. On one hand, our study confirmed that the 17-item 3-factor structure of ZBI from the UK study [13] is suitable for informal caregivers of PWD in Singapore. The first factor is ‘impact on caregiver life’, includes the impact of caregiving on caregiver’s privacy, finance, personal health, family and work, social life, etc. The second factor is ‘uncertainty over future’, this is more about the concerns regarding what the future would hold for both the caregiver and the PWD. The third factor is ‘frustration/embarrassment’, it is about the emotional reaction caregivers might have while caring for the PWD. On the other hand, the latent class analysis revealed that the current sample could be divided into three mutually exclusive caregiving burden classes: high, medium, and low; and the caregiving burden pattern showed that caregivers tended to have similar ratings on all domains of ZBI. In other words, if caregivers have higher burden on one domain such as impact on caregiver life, they are likely to experience higher burden on the other two domains as well. This is different from our previous assumption that informal caregivers might experience different levels of burden on different domains of caregiving burden. Nonetheless, these two findings together justify that more attention should be given to caregivers experiencing an overall high caregiving burden to provide them with more support.

Some factors were found to be significantly correlated with both the latent factors and the latent classes of ZBI, and they are mainly caregiving related variables, including caregivers’ self-rated health, weekly caregiving hours, and PWD’s ADL and MBP. This is similar to a recent study suggesting the importance of caregiving related factors over socio-demographics in predicting caregiving burden [48]. The multiple linear regression analyses found that MBP is the only factor which significantly correlated with all three factors of ZBI. This is consistent with findings from the previous UK study which suggested significant correlations between PWD’s behaviour changes and their caregiving burden [13]. Furthermore, our study also suggested that MBP is the only factor which is significantly correlated with caregiving burden group, evidenced by the fact that caregivers who provided care for PWD with more MBP were more likely to be in a higher caregiving burden group. Together this suggests the primary role of PWD’s memory and behavioural problems such as repeating the same questions or aggression in predicting caregiving burden [49]. Caregivers’ self-rated health was found to be able to differentiate between high- vs. medium- and low caregiving burden groups, with caregivers who fall under the high burden group more likely to have fair and poor health. Previous studies had explored the effect of caregiving burden in predicting caregivers’ health [50, 51]; however, this effect could be bidirectional as poor health of caregivers also make their caregiving more challenging. From our study, caregiver’s self-rated health might exacerbate caregivers’ feeling of impact on their life and uncertainty over the future. However, the cross-sectional design of this study precludes us from establishing any causal relationships, future longitudinal studies are needed. Similarly, weekly hours spent in caregiving was identified as one of the influencing factors of caregiving burden [49], and our study further suggested that it might be through influencing the perceived impact on caregivers’ life. Last but not least, PWD with higher ADL would cause more burden to caregivers in terms of impact on their life, and caregivers caring for PWD with higher ADL were more likely to be in the high- vs. low caregiving burden group.

In contrast, certain factors were found to be either correlated only with the latent factors or only with the latent classes of ZBI. Specifically, caregivers’ age, ethnicity and relationship to PWD were found to be significantly correlated with the latent factors of ZBI; while education was significantly correlated with the caregiving burden groups. Interestingly, we found that age is negatively associated with caregivers’ perceived burden on uncertainty over future and frustration/embarrassment, and spouse caregivers were more likely to report higher scores on uncertainty over future compared to adult-child and other relationship caregivers. This finding differed from the UK study [13], which suggested that age was only negatively correlated with impact on caregiver life and adult-child caregivers felt particularly burdened on uncertainty over the future. There are several possibilities. Firstly, it could be due to the fact that we controlled for variables including self-rated health in our analysis and self-rated health usually declines with age [52]. In this case, self-rated health might work as a mediator between age and impact on caregiver life, and fully mediated the relationship. Secondly, it could also be that caregivers with older age might experience more positivity in caregiving [2] and such feelings buffer the impact of caregiving on uncertainty and frustrations. Last but not least, it could be due to cultural differences, more specifically filial piety in the Asian societies [53]. Filial piety alone gives a huge motivation for adult-child to take care of the PWDs, and studies also suggested that it might serve as a protective factor on caregiving burden [54, 55]. We are uncertain about what exactly caused the difference in the findings from the UK study, hence more research is needed to further investigate this topic. Compared to Chinese caregivers, Malay caregivers reported significantly lower scores on impact on caregiver life. This is consistent with a previous study in Malaysia which also suggested that Malays caregivers tend to experience lower caregiving burden compared to Chinese caregivers [56]. Lastly, similar to other studies [15, 57], education level was not significantly correlated with any ZBI factors in our study. However, our study also suggested that higher education level was associated with higher odds of being in the high- compared to the low caregiving burden group, indicating that though education had no impact on any of the factors of ZBI, it can still affect level of caregiving burden. One possibility is that caregivers with higher levels of education might have a stronger aversion towards restrictions of autonomy as caring for PWD usually involves significant amount of time and efforts [58], which in turn led to their escalated mental burden.

Findings from this study have practical implications. Firstly, the current study provided a holistic picture of caregiving burden among informal dementia caregivers, suggesting that it would be useful to study caregiving burdens by classifying it into low, medium and high levels. Caregivers experiencing higher burden on one domain are also likely to experience higher burden on other domains. As a result, it is reasonable to focus on caregivers experiencing an overall high caregiving burden to provide more support. From this point of view, a short screener like the 6-item version ZBI [59] might be helpful. Secondly, our study further emphasizes the importance of PWD’s memory and behaviour problems on caregiving burden. This again implies the importance of early diagnosis of dementia as it provides the opportunity for earlier treatment which may slow down the disease progression [60], and the importance of providing respite services to relieve their caregiving burden [61]. Lastly, although correlates on latent factors and latent classes of ZBI differed from each other, it would still be worthwhile to study them simultaneously as all the significant correlates are important to understand caregiving burden and in identifying caregivers at higher risks.

There are several strengths of this study. First of all, unlike the 4-factor structure of ZBI identified from previous studies among informal dementia caregivers in Singapore [14, 15], our study suggested the 3-factor structure of ZBI from UK dementia caregiver [13] is applicable locally. As all factors under this structure contain more than three items, this factor structure is arguably more stable [16]. Secondly, this is the first study which uses both a variable-centered and a person-centered analytic approach to understand caregiving burden among informal dementia caregivers. The advantage of such a combination is that it not only explains the relationships between variables of interest, but also captures the heterogeneity of the study sample [17], thus providing a more objective picture of the investigated topic. Lastly, this is also the first study that explored the similarity and differences in the significant correlates of latent factors and latent classes of ZBI, and the findings further supports the importance of caregiving related variables especially PWD’s memory and behaviour problems in caregiving burden.

We should also bear the following limitations in mind. Firstly, the caregivers were recruited through convenience sampling and were thus, a self-selected sample, which might affect the generalizability of the study findings. Nonetheless, findings from our study are mostly consistent with previous studies based on our comparisons. Secondly, data were collected through self-reported measures in the format of interviewer-administered questionnaire, this method might lead to recall bias [62] and social desirability bias [62, 63]. Thirdly, though RMBPC has three subscales, only two on memory and behavioral problems were used in the current study. It is possible that PWD's depressive symptoms might also affect caregiving burden. Future studies can further explore this topic. Additionally, due to the relatively small sample of our study, multinomial logistic regression was conducted separately from the latent class analysis modeling. This 3-step approach in fitting LCA might lead to less accurate predictions of the associations [41]. Lastly, the cross-sectional design precluded us from drawing any conclusions on the causal-relationships, especially on the relationship between caregivers’ self-rated health and caregiving burden. As a result, longitudinal studies such as those measuring the health status and caregiving burden of caregivers before and after they take over the caregiving responsibilities are needed to further test our hypotheses.

## Conclusions

The current study found that the 3-factor structure of ZBI from the UK study was applicable among informal dementia caregivers in Singapore. From the comparisons between the UK study and our study, we infer that culture might affect how caregivers perceive caregiving burden. Our study also extended previous research on ZBI by using a latent class analysis, which revealed that there were three distinct caregiving burden classes. Through the combination of the variable-centered and person-centered analytical approaches, we found that informal dementia caregivers tend to have similar ratings on different domains of caregiving burden. This finding justifies that more attention should be given to support caregivers who experience an overall high burden. Research is needed to test if this finding is also applicable among dementia caregivers elsewhere. Caregiving related variables especially PWD’s memory and behaviour problems played a very important role in caregiving burden. Future research may wish to explore the types of support needed by informal dementia caregivers, especially among those experiencing high burden.

## Data Availability

All individual data from this study resides with Office of Research, Institute of Mental Health. Data is not available for online access, however readers who wish to gain access to the data can write to the Clinical Research Committee, Institute of Mental Health/Woodbridge Hospital Secretariat at IMHRESEARCH@imh.com.sg. Access can be granted subject to the Institutional Review Board (IRB) and the research collaborative agreement guidelines. This is a requirement mandated for this research study by our IRB and funders.

## Abbreviations

PWD: Persons with dementia
ZBI: Zarit Buren Interview
ADL: Activities of Daily Living Scale
IADL: Instrumental Activities of Daily Living Scale
MBP: Memory and behaviour problems
RMBPC: Revised Memory and Behaviour Problems Checklist
SD: Standard deviation
CFA: Confirmatory factor analysis
CFI: Comparative fit index
TLI: Tucker-Lewis index
RMSEA: The root mean square error of approximation
LCA: Latent class analysis
AIC: The Akaike Information Criteria
BIC: The Bayesian Information Criteria
cAIC: The consistent Akaike Information Criteria
OR: Odds ratio
